# Facilitation of Function and Manipulation Knowledge of Tools Using Transcranial Direct Current Stimulation (tDCS)

**DOI:** 10.3389/fnint.2017.00037

**Published:** 2018-01-04

**Authors:** Ryo Ishibashi, Tatsuya Mima, Hidenao Fukuyama, Gorana Pobric

**Affiliations:** ^1^Neuroscience and Aphasia Research Unit, Division of Neuroscience and Experimental Psychology, University of Manchester, Manchester, United Kingdom; ^2^Smart Aging Research Center, Tohoku University, Sendai, Japan; ^3^Graduate School of Core Ethics and Frontier Sciences, Ritsumeikan University, Kyoto, Japan; ^4^Human Brain Research Center, Graduate School of Medicine, Kyoto University, Kyoto, Japan; ^5^Intelligent Robotics Institute, Beijing Institute of Technology, Beijing, China

**Keywords:** transcranial direct current stimulation (tDCS), cognitive enhancement, semantics, praxis, tool use

## Abstract

Using a variety of tools is a common and essential component of modern human life. Patients with brain damage or neurological disorders frequently have cognitive deficits in their recognition and manipulation of tools. In this study, we focused on improving tool-related cognition using transcranial direct current stimulation (tDCS). Converging evidence from neuropsychology, neuroimaging and non- invasive brain stimulation has identified the anterior temporal lobe (ATL) and inferior parietal lobule (IPL) as brain regions supporting action semantics. We observed enhanced performance in tool cognition with anodal tDCS over ATL and IPL in two cognitive tasks that require rapid access to semantic knowledge about the function or manipulation of common tools. ATL stimulation improved access to both function and manipulation knowledge of tools. The effect of IPL stimulation showed a trend toward better manipulation judgments. Our findings support previous studies of tool semantics and provide a novel approach for manipulation of underlying circuits.

## Introduction

Tool use is one of the most remarkable cognitive skills in humans. Modern life involves the use of a wide variety of manmade items, from ceramic kitchen scissors to smartphones. We can recognize familiar tools at once and perform the proper action for each of them with ease. How the human brain implements this ability still remains to be fully explained. At least two cognitive components seem involved: recognition of the tool’s function and correct manipulation. To purposefully use a particular tool, one should be able to correctly identify its function and retrieve how to manipulate it. These two components (recognition of tool function and memory of manipulation) may be underpinned by separable brain regions. The earliest reports supporting this argument come from neuropsychology. Here, tool-use deficits have been observed after damage of the left parietal lobe, often described with the term “apraxia”, especially as “ideational apraxia” (Heilman and Rothi, 1993) or “conceptual apraxia” (Ochipa et al., 1992; Zadikoff and Lang, 2005). Patients with apraxia manifest difficulty in using objects properly, despite preserved physical function of the limbs (Gonzalez Rothi and Heilman, 1997). Investigations revealed that patients with ideational/conceptual apraxia have compromised knowledge of how to move their body parts to use familiar tools due to infarction at/around left inferior parietal lobule (IPL) after stroke (Buxbaum et al., 2000; Buxbaum and Saffran, 2002; Rosci et al., 2003). Similarly, Gainotti (2000, 2004) has suggested that lesions in left fronto-parietal and sensory cortices lead to category–specific deficits in the representation of tools and other manmade items.

While these studies on apraxia report deficits in knowledge of tool-use actions, studies of semantic dementia (SD) have reported deficits in knowledge about tool function. Patients with SD suffer from symptoms reflecting degraded conceptual knowledge of objects. This is believed to result from progressive degeneration of the anterior temporal lobe (ATL). Studies suggest that the severity of their semantic deficit correlates with the extent of atrophy in bilateral ATLs (Mummery et al., 2000; Nestor et al., 2006). Neuropsychological investigations of verbal/non-verbal cognitive function in patients with SD revealed that they have compromised knowledge of how to use familiar objects in purposeful ways (Bozeat et al., 2000, 2002). In a longitudinal study of two SD cases over 4 years, Coccia et al. (2004) also showed a parallel decline in general semantics and object-use ability. This indicates that the tool-use deficit in SD patients stems from their compromised comprehension of tools themselves, including in which context a tool should be used. These studies on SD indicate that general semantic knowledge, including the function of each object, contributes to daily object-use ability and that the semantic comprehension of objects most likely is supported by the ATLs in both hemispheres.

Several neuroimaging studies with healthy participants reported distinctive activations associated with object function and action in the ATL and IPL regions, respectively. Kellenbach et al. (2003) used positron emission tomography (PET) to explore object-related cortical regions associated with function or action of familiar tools. Following visual presentation of a familiar tool, participants answered one of various questions that tapped into their knowledge of the tool’s function (e.g., “Is the object used to attach or hold objects together?”) or action (e.g., “Does using the object involve a back-and-forth action?”). Kellenbach et al. (2003) found increased activation in a region around the left IPL (with a peak at the intraparietal sulcus) when the participants were making decisions about actions with objects. Boronat et al. (2005) found a similar activation in the left parietal lobe using functional magnetic resonance imaging (fMRI). Their task involved presentation of object pairs that could have the same function (e.g., matches and lighter) or action (e.g., piano and computer keyboard). Participants were asked to judge if the pair had a semantic association in terms of function or action. Comparison of the two task conditions revealed higher activation in the left IPL for action-matching relative to function-matching. A similar task was used by Canessa et al. (2008). They reported contrasting activations in IPL and inferior temporal cortex in the left hemisphere. In their object matching task, participants judged the semantic relevance of object pairs based on the context of object use (used in the kitchen, gardening, etc.), as well as the physical action employed to use the tool. The data revealed significantly higher activation in the left IPL for action than for context judgment. In the left posterior middle temporal gyrus there was higher activation for context than for action judgment. This implies dissociable roles of the left IPL and the temporal lobe.

Evidence for involvement of IPL and ATL regions is further supported by recent studies using non-invasive brain stimulation. Pobric et al. (2010) applied 1-Hz repetitive transcranial magnetic stimulation (rTMS) to inhibit focal cortical activities in the left ATL/IPL in healthy participants before asking them to perform object naming. Results showed slightly delayed responses for manipulable items after rTMS to IPL, while ATL stimulation slowed naming of all types of concepts including living and non-living items. In our previous study (Ishibashi et al., 2011), we used the same rTMS-protocol as Pobric et al. (2010) to examine the roles of left ATL and IPL in tool cognition. In the study, we asked participants to perform a tool-matching task based on the tool’s function or manipulation (action). Object pairs with similar actions (e.g., scissors and pliers) had to be chosen in the manipulation judgment task, while pairs with similar functions (e.g., scissors and knife) were to be selected in the function judgment task. The results showed increased response times (RT) for function matching after rTMS on ATL, as well as increased RT for manipulation matching after rTMS on IPL. A similar dissociation between temporal and parietal lobes was observed by Andres et al. (2013) with tasks requiring recall of various contexts of using particular tools or of the hand configuration in using them. They found slower RT in the hand configuration task after rTMS on the left SMG (the rostral part of IPL). These results indicate separable cognitive processing in IPL and ATL by demonstrating the causal relationship between magnetic stimulation and the performance of cognitive tasks.

Though the above findings with rTMS demonstrate crucial roles for the ATL and IPL regions in tool recognition and use, it is still unclear whether human tool cognition *can be improved* by some intervention into these two areas using non-invasive brain stimulation. The rTMS protocol described above is effective to induce transient reduction of activity in a particular region of the brain (Walsh and Rushworth, 1999). A more practical question would be whether increasing the activity in these areas improves tool cognition performance. Answering this question should be of great interest to clinicians and caregivers of patients suffering from major deficits in daily tool-use ability. Testing this possibility requires another method of non-invasive stimulation that is capable of up-modulating the cortical activity of an area: transcranial direct current stimulation (tDCS). This method uses a weak (1–2 mA) direct current on particular cerebral areas of interest. Applying continuous current to the brain through the skull modulates the membrane potential of neurons under the electrodes in a polarity-dependent way (Nitsche and Paulus, 2000, 2011). Anodal stimulation depolarizes the membrane potential, thus resulting in an increased firing rate in the area, while cathodal stimulation hyperpolarizes it, leading to a decreased firing rate. Therefore, the application of electrical currents could influence cortical excitability and affect behavior (Bestmann et al., 2015). Some attempts have been already made to explore its application in alleviating cognitive deficits after stroke (Pollock et al., 2014) or progressive neuro-degenerative disorders (Hung et al., 2017). Based on these promising reports, we explored the potential facilitative effect of anodal tDCS in healthy volunteers. Following on from previous findings, we focused on the question whether stimulating the left ATL or IPL improves recognition of tool function/action. In line with previous studies (Pobric et al., 2010; Ishibashi et al., 2011), we expected to observe a modality-general facilitation effect by ATL-tDCS and an action-specific facilitation effect by IPL-tDCS.

## Materials and Methods

### Participants

Eighteen students from Kyoto University (21–27 years old, mean age = 22.3, 13 males) took part in this study. All had normal or corrected-to-normal vision. This study was approved by the Ethics Committee at the Graduate School and Faculty of Medicine, Kyoto University (reference code: C800). All participants provided written consent before starting the study in accordance with the Declaration of Helsinki.

### Stimuli

**Figure 1:**
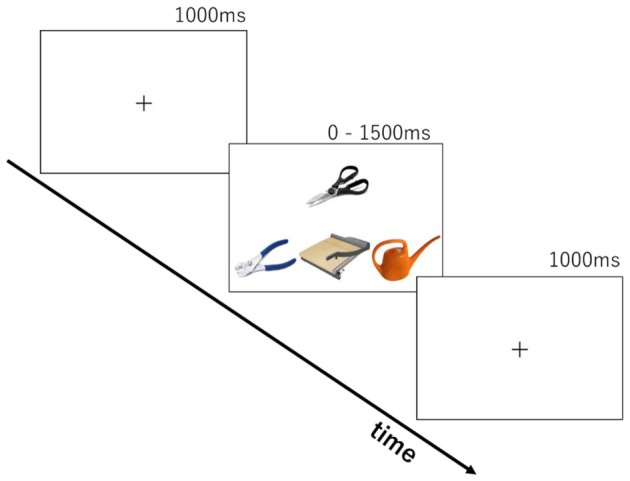
Stimulus presentation in the two cognitive tasks.

The stimuli were based on tasks from clinical investigations of tool use knowledge in SD patients (Hodges et al., 2000; Bozeat et al., 2002; Corbett et al., 2009). Two-hundred tool images collected from the internet were used. The tools were organized into 90 sets of four images by sampling with replacement. Thirty-three items were used for 10 practice trials and the remaining 167 items were used to compose 80 trials for real testing. Each trial (see Figure 1) consisted of a probe object (e.g., scissors), a function target that had the same/similar function as the probe (e.g., guillotine), a manipulation target that is manipulated in the same/similar ways as the probe (e.g., pliers) and a foil (e.g., whisk).

### Task

We developed two tasks to tap into knowledge of either tool function or tool manipulation. The same stimulus set was used in both function and manipulation tasks. Each trial consisted of the presentation of four photos of tools (see Figure 1) for up to 1500 ms following a 1000 ms presentation of a fixation cross. The probe object (e.g., scissors) was presented above the other three. In the function matching task, participants were asked to choose the tool from the three objects with the same/similar function as the probe (e.g., guillotine, for cutting). In the manipulation matching task, they were asked to choose the tool with the same/similar way of manipulation (e.g., pliers, by gripping handles with one hand). Ten trials were chosen beforehand for practice. After two cycles of practice trials for each task, the participants performed 160 trials in total (80 for each task). There was a self-paced rest after every 40 trials. The order of tasks was counterbalanced across participants. Participants pressed the left-, down-, or right-arrow keys on the keyboard, corresponding to the choice of an object at left, middle, or right. Stimuli were presented until response for a maximum of 1500 ms. A 1000 ms blank screen was inserted before starting the next trial. The time limit of 1500 ms was introduced to produce enough cognitive load for the young, healthy participants and increase task difficulty. E-prime 2.0 (Psychology Software Tools Inc.) running on a laptop PC was used for stimulus presentation and recording of participants’ responses.

### Procedure

**Figure 2:**
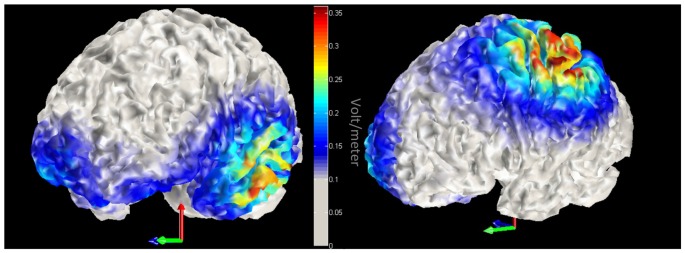
Model of transcranial direct current stimulation (tDCS) current. Red-yellow colors indicate increased magnitude of the total electric field due to tDCS. Left panel displays left anterior temporal lobe (ATL) stimulation, while the right panel highlights stimulation within the left inferior parietal lobule (IPL).

We employed a within participant design. Participants took part in three sessions on separate days with at least a 5-day interval between them. In each session a different brain region was stimulated: left ATL, left IPL and sham in counterbalanced order. A 3 × 3 cm anodal electrode was put over position FT7 (according to the international 10-10 EEG system) for ATL stimulation, or CP3 for IPL stimulation, and a 5 × 5 cm cathodal electrode (reference) was placed on the right of the forehead (1 cm below FP2). The site of sham stimulation was randomly chosen. Half of the participants received sham stimulation over left ATL; the other half over left IPL. The sponge electrodes were soaked with saline prior to each session to increase electrical conductance. A NeuroConn DC Stimulator Plus (NeuroCare Inc.) delivered the direct current stimulation. Duration of stimulation was synchronized with task duration (avg = 17.8 min). Stimulation intensity was set initially at 2.0 mA. If the participant perceived the stimulation as uncomfortable, the current was lowered by steps of 0.1 mA. To ensure that participants were familiar with the stimuli, all 167 pictures of tools and their names were presented for 1000 ms each. Subsequently, participants went through a practice block that contained 10 trials for each task. After practice, the DC stimulation was initiated. Stimulation was set to reach the designated current with an 8 s ramp-up period and 8 s ramp-down period. In the sham stimulation condition, the electric current lasted 30 s. Four blocks of 40 trials were administered, counterbalanced by task (tool function/manipulation). Participants took self-paced rests between the blocks. Using these tDCS parameters, we modeled the magnitude of the total electric field due to stimulation with COMETS (Jung et al., 2013). The model provided evidence that the tDCS electric field was largest in the left ventral ATL and left IPL (please see Figure 2).

## Results

As five participants reported an uncomfortable tingling feeling on electrodes during the initial phase of stimulation, the level of stimulation was downregulated by steps of 0.1 mA in 8 of 54 sessions. The average current intensity across all participants remained at 1.9 mA.

**Figure 3:**
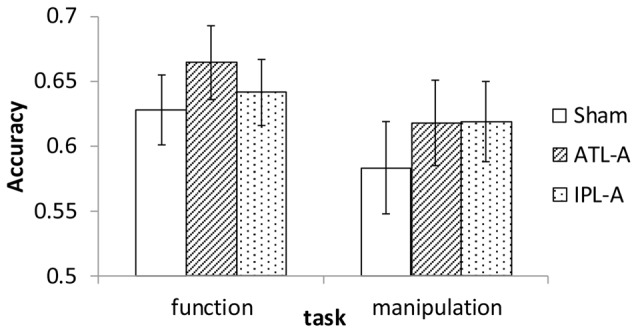
Accuracy in sham, ATL and IPL conditions. Error bars indicate standard error.

Since the experiment was designed to examine accuracy data in each task within the imposed limited time for each trial (1500 ms), we did not put a major effort into analyzing RT. Mean RT in each condition was calculated and almost the same across all conditions (1123–1145 ms). Accuracy data were encoded binomially (success = 1, failure = 0) for use in the analyses. Mean accuracy in each task/tDCS condition is shown in Figure 3. Compared to sham stimulation, participants showed better performance with ATL stimulation in the function task. In the manipulation task, performance was better in both the ATL and the IPL stimulation condition compared to sham. We conducted a series of logistic mixed-effect model analyses using the *lme4* package in R^1^. Linear mixed effects models account for within subject correlations more optimally than Analysis of Variance (ANOVA). In order to estimate the effects of tDCS, the sham condition was used as a reference level in the regression, which allowed estimating regression coefficients for ATL and IPL stimulation. The order of the sessions (1st, 2nd, or 3rd) was also included to control for the effect of participants’ adaptation to the tasks. First, we conducted an analysis including all possible main effects and interactions. The involved fixed effects were task (function/manipulation), stimulation (ATL, IPL, sham), session order (1st, 2nd or 3rd). The random effects were participant and trial. Interaction between task and stimulation was also included in the model as a fixed-effect term. The results showed a significant main effect of ATL stimulation (*z* = 2.35, *p* < 0.05), but no significant effect of IPL stimulation (*z* = 0.71, *p* > 0.10). Interaction effects between task and stimulation were not significant (*z* = −0.11 in ATL and *z* = 0.939 in IPL, *p* > 0.10), nor were the interactions between session order and task (*z* < 1.38, *p* > 0.10).

**Figure 4:**
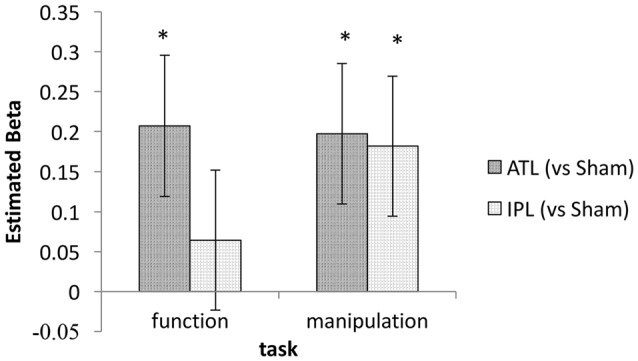
Estimated regression coefficient values for the tDCS effect (ATL/IPL vs. sham). Error bars indicate estimated standard deviation. *Indicates statistically significant deviation from zero (*p* < 0.05).

Based on our own previous neurostimulation studies (Pobric et al., 2010; Ishibashi et al., 2011) and extensive literature (Buxbaum et al., 2000; Buxbaum and Saffran, 2002; Rosci et al., 2003), we had a strong expectation to find an interaction between stimulation and task. We therefore, explored the effects of tDCS on ATL and IPL in the two tasks separately. This task-wise mixed-effect model analyses found significant effects of ATL stimulation in both function and manipulation tasks (*z* = 2.35 and 2.25 respectively, *p* < 0.05). The effect of IPL stimulation was significant for the manipulation task (*z* = 2.08, *p* < 0.05), but not for the function task (*z* = 0.73, *p* > 0.10). Estimated beta values for ATL and IPL in each task are shown in Figure 4. This exploratory analysis suggests that an interaction between task and stimulation site may exist, since the beta values conform to the expected pattern.

## Discussion

In the present study, we found an enhancing effect of tDCS applied over the left ATL in semantic tasks requiring knowledge of function or action of familiar tools. The effect of ATL stimulation was task-general (both manipulation and action matching). Our exploratory analysis revealed that there may be a task-specific effect of IPL stimulation for the manipulation task. The trend towards a task-dependent pattern of the stimulation effect partially corresponds with our previous rTMS study (Ishibashi et al., 2011), in which we showed an effect of ATL stimulation in a tool function task and an effect of IPL stimulation in an action-judgment task. Our current study demonstrates effects of anodal tDCS on retrieving the semantic information necessary for human tool use. The task-invariant facilitation by anodal ATL-tDCS is particularly important when considering the potential outcome of applying non-invasive stimulation to this region. The left and right ATLs have been discussed in association with modality-general processing of various concepts. Neuropsychological investigations of patients with SD provide converging evidence for the importance of these regions for semantic cognition in various contexts, such as comprehension of speech or written words or naming pictures of familiar items (Jefferies and Lambon Ralph, 2006; Crutch and Warrington, 2008). The focal atrophy in these regions is believed to cause deterioration in the patients’ ability to deal with the environment in a meaningful way. While dysfunction in a verbal-linguistic context, such as difficulty in word comprehension and anomia, is prominent in SD patients, non-verbal semantic impairments including tool-use deficit were also reported in the last few decades. The ability of SD patients to use familiar objects was investigated by Hodges et al. (2000). They found that patients with SD indeed performed below the normal range in tool recognition/use. Importantly, performance was commensurate with the degree of degradation in semantics measured with picture naming, a category fluency test, word-picture matching and the Pyramid and Palm Trees Test (Howard and Patterson, 1992). In addition, there was a strong by-item correlation between tool-naming and tool-use scores, which indicates item-by-item consistency in performance (i.e., inability to name/recognize a tool is accompanied by an inability to use it). This parallelism of conceptual knowledge and action knowledge about tools is also indicated by a longitudinal study of SD patients by Coccia et al. (2004). Assessments of the verbal and non-verbal semantic competency of two SD cases over 4 years, found that tool-use ability declined in parallel with the degradation of the patients’ general semantics. Other studies also report an association between semantic memory and tool-use ability in SD (Hamanaka et al., 1996; Hodges et al., 1999). Taken together, these reports suggest that ATL supports general object semantics and hence that damage to this area can lead to degraded ability of both understanding and making use of common tools. The current finding of enhanced tool cognition by facilitative electrical stimulation over ATL does not only corroborate this view, but also identifies the possibility of modulating the activity of the “semantic hub” to improve recognition and use of common tools, an ability essential in modern human life.

The increased accuracy in our tool judgment task during ATL-tDCS is also in line with our own previous findings. Pobric et al. (2010) reported category-invariant slowing of naming by applying rTMS to ATL, while IPL stimulation yielded effects selectively for manipulable, non-living items only, demonstrating the generality of the ATL contribution to object naming. The task-general effect of ATL stimulation is essentially the same in the current experiment. Our previous TMS study with the same type of tasks (Ishibashi et al., 2011) showed that function knowledge about tools are temporarily hampered by repetitive TMS on the left ATL, which indicated the role of this region in representing object functions. The rTMS study, however, did not find an effect of ATL stimulation on tool-action judgments. One explanation of this apparent discrepancy is that the difficulty of tool action judgments was much higher in this study, due to the imposed time limit. The task was designed to be difficult enough for healthy participants to make sufficient errors when forced to respond in a short time. Due to the tight time restrictions in the current study the mean accuracy without DC stimulation was about 0.60, providing a sufficient margin for improvement. Although it is not reasonable to compare RT and accuracy measures across different experiments, the sensitivity of the current tasks to improvement may have been a major factor contributing to the difference between these stimulation studies. The present results suggest that the accuracy of tasks in relatively difficult setting, combined with a sensible statistical method, can provide an appropriate measure to test the effect of the transcranial DC stimulation.

The current results also suggest at a possible contribution of the IPL region to judging the manipulation of familiar objects. The task-wise GLMM analyses suggested that the effect of IPL stimulation was much larger for the manipulation-than for function-judgment task. The direction of this result agrees with the semantic hub and spoke model. Judging manipulation is arguably more directly linked to representations of body movement and thus should be more modality-specific than function attributes. In fact, as discussed in the “Introduction” section, stroke patients with infarction in the left parietal cortex often manifest symptoms of ideational apraxia, a neuropsychological condition characterized by an inability to manipulate familiar objects in proper ways. Furthermore, some seminal work on post-stroke cognitive deficits report that patients with the left parietal damage show category-specific semantic impairment for tools. Warrington and McCarthy (1983, 1987) reported several patients with left temporo-parietal or fronto-parietal infarction whose recognition of manmade objects was selectively impaired while sparing the ability to recognize animals. Gainotti (2000) reviewed reports of 47 post-stroke patients and concluded that damage in the dorso-lateral regions of the left hemisphere (ranging from temporo-parietal to frontal areas) leads to category-specific semantic impairment for manmade artifacts. This notion is strengthened by another meta-analysis by the same author (Gainotti, 2004) which showed that patients with focal damage in temporal lobes (with spared fronto-parietal cortex) are generally better in recognizing artifacts than living things. This line of focused investigations on the correlation between the pattern of brain damage and category specificity in semantic deficits supports the contention that a part in the left parietal lobe contributes to composing the core information of the manipulable, manmade objects. A candidate for this role is the IPL, due to its location on the course of occipital-visual to frontal-motor areas (Zhang and Li, 2014). According to a recent theory about multiple visual pathways to process object information (Buxbaum and Kalénine, 2010), IPL is well located at the end of a cortical pathway (ventro-dorsal pathway) that contributes to retrieving well-learned manipulative actions to use various common objects, such as movement of arm, wrist and fingers. The implied gradation of the tDCS effect favoring the manipulation over the function task is in line with this hypothesis.

A possible alternative account for the role of IPL, however, would be that this area computes tool-body interaction anew rather than that it represents and stores information about tool-use actions. In fact, the parietal lobe in general has long been argued to convert visual information into motor representations to allow body movements towards tools (Mühlau et al., 2005; Molenberghs et al., 2009). A voxel-based lesion-symptom mapping (VLSM) study with 34 patients with a cerebrovascular accident in the left hemisphere (Goldenberg and Spatt, 2009) found that damage in the left parietal lobe correlated more with performance in novel object use than in familiar tool use. However, considering how heterogeneous the overall functions of IPL are, it is possible that the areas for representation and computation of object-use actions are close yet separable. In fact, in the VLSM study by Goldenberg and Spatt (2009), the area with the highest rate of overlapping lesion voxels associated with novel-tool actions was located in a part of IPS, posterior to the SMG/BA40 region targeted for stimulation in the current study. This area was also reported in other neuroimaging reports that trained the use of novel tools. Weisberg et al. (2007) had healthy volunteers learn to use 16 novel objects that did not have any resemblance to common tools. They found that a part of the left IPS showed higher activation after three training sessions (amounting to 4.5 h) of learning proper use of the novel objects. Similar results were found in other studies that trained novel tool use by observation of the manipulation of items (Bellebaum et al., 2013; Rüther et al., 2014). Arguably, novel repertoires of tool-use actions are computed in an area around posterior IPS and later transferred into SMG for long-term storage. How the novel set of actions for unfamiliar tools are created and later consolidated in different IPL subregions is still to be elucidated.

The positive outcome of anodal tDCS for cognitive processes related to tools is potentially of great importance for practical applications. At present, the application of tDCS as an auxiliary method for treatment after stroke has already shown some positive effects for the motor function of upper limbs (Pollock et al., 2014; see Lüdemann-Podubecká et al., 2014 for a review and lower limbs (van Asseldonk and Boonstra, 2016). Psychiatric disorders such as schizophrenia and depression are also major targets of tDCS interventions (Mondino et al., 2014; Pondé et al., 2017). Cognitive-linguistic impairments such as stroke aphasia are also becoming a potential target (Baker et al., 2010; Marangolo et al., 2016; see ALHarbi et al., 2017 for a review). However, there are still only a few reports of tDCS effects on recovery from apraxic symptoms (Bianchi et al., 2015; Bolognini et al., 2015). These reports focused on production of gestures but not on transitive tool-use actions. In light of the present finding that indicates a possible enhancing effect of tDCS for tool-use knowledge, this line of research with patients is worth pursuing. As to the general semantic deficits caused by SD, anodal tDCS of the temporo-parietal cortex (P3 in the international 10-10 system) combined with cognitive therapy has been proven to slow down the progress of symptoms, at least for words trained during stimulation (Hung et al., 2017). Testing of healthy volunteers with semantic tasks has accumulated evidence of the effect of tDCS on various regions in the brain (for a review, see Joyal and Fecteau, 2016). The majority of studies with tDCS aiming to modulate semantic cognition stimulated frontal areas, but essentially no tDCS study so far focused specifically on the anterior part of the temporal lobe (but see Penolazzi et al., 2013 for a montage-dependent improvement of word fluency by stimulation of the fronto-temporal area). As this region is frequently mentioned as the central neural circuit to bind sensori-motor information to create semantic representations, the possible enhancing effect of tDCS may deserve further investigation in future studies.

It should be noted that this research had some limitations. The exact mechanism of tDCS is still not known (Bestmann et al., 2015). As any information should be represented as a “pattern” of cortical activation in a particular region/multiple areas distributed in the brain, it is possible that only the representations that could benefit from heightened activations in ATL/IPL were enhanced in the current experiment. This could be also one of the reasons for the relatively weak effect of tDCS which in the present study led to the lack of conclusive evidence for task specific IPL stimulation.

## Conclusion

We report that anodal tDCS on ATL/IPL improves cognitive function associated with familiar objects. The task-general effect by ATL stimulation and the trend towards a task specific effect of stimulation over IPL are consistent with the hub-and-spoke hypothesis of human semantic processing. As the effect was facilitatory in both tasks, this method of non-invasive stimulation may have potential as a therapeutic intervention for rehabilitation after stroke or for treatments of progressive neurological disorders that affect semantic memory for common objects.

## Author Contributions

RI and GP conceived the idea, designed the study, interpreted the data and wrote the manuscript. RI executed the study and carried out data analyses. TM and HF contributed to data interpretation.

## Conflict of Interest Statement

The authors declare that the research was conducted in the absence of any commercial or financial relationships that could be construed as a potential conflict of interest.

## Footnotes

^1^https://cran.r-project.org/web/packages/lme4/index.html
